# Generation of Advanced Blood–Brain Barrier Spheroids Using Human‐Induced Pluripotent Stem Cell‐Derived Brain Capillary Endothelial‐Like Cells

**DOI:** 10.1002/adbi.202400442

**Published:** 2025-02-06

**Authors:** Sanjana Mathew‐Schmitt, Sabrina Oerter, Evelin Reitenbach, Sabine Gätzner, Alevtina Höchner, Heinz‐Georg Jahnke, Jörg Piontek, Winfried Neuhaus, Andreas Brachner, Marco Metzger, Antje Appelt‐Menzel

**Affiliations:** ^1^ Chair Tissue Engineering and Regenerative Medicine (TERM) University Hospital Würzburg 97070 Würzburg Germany; ^2^ Fraunhofer Institute for Silicate Research ISC Translational Centre Regenerative Therapies (TLC‐RT) 97070 Würzburg Germany; ^3^ Biotechnological‐Biomedical Center (BBZ) University of Leipzig 04103 Leipzig Germany; ^4^ Clinical Physiology/Nutritional Medicine Department of Gastroenterology Rheumatology and Infectious Diseases Charité–Universitätsmedizin Berlin 12203 Berlin Germany; ^5^ AIT Austrian Institute of Technology GmbH Centre Health and Bioresources, Competence Unit Molecular Diagnostics Vienna 1210 Austria; ^6^ Department of Medicine Faculty Dentistry and Medicine Private Danube University Krems 3500 Austria

**Keywords:** blood–brain barrier (BBB), hiPSC‐derived brain capillary endothelial‐like cells (iBCECs), human‐induced pluripotent stem cells (hiPSCs), neurovascular unit (NVU), self‐assembled BBB spheroids

## Abstract

Extensively studied blood–brain barrier (BBB) in‐vitro models are established on 2D cell culture inserts. However, they do not accurately represent 3D in‐vivo microenvironments due to lack of direct neurovascular unit cellular contacts. Here, the establishment and characterization of a self‐assembled 3D BBB spheroid model using human‐induced pluripotent stem cell (hiPSC)‐derived brain capillary endothelial‐like cells (iBCECs) in combination with primary human astrocytes (ACs) and pericytes (PCs) are reported. This investigation compares 3D spheroids with 2D mono‐cultured iBCECs derived from two different hiPSC lines and two differentiation strategies. It is observed that spheroid properties vary depending on the differentiation strategy or type of hiPSC line applied for model generation. However, spheroids demonstrate in‐vivo like tight junction ultrastructure and, in comparison to 2D models, higher transcript expression of BBB specific genes. Furthermore, they possess characteristic barrier integrity, barrier functionality, and protein expression. It is inferred that hiPSC‐derived BBB spheroids hold a strong potential as a reliable future BBB in‐vitro test system.

## Graphical abstract

In this article, the establishment and characterization of a self‐assembled 3D BBB spheroid model using human induced pluripotent stem cell (hiPSC)‐derived and primary cells is reported. Spheroids demonstrate in‐vivo like tight junction ultrastructure and, in comparison to 2D mono‐cultures, higher transcript expression of BBB specific genes. HiPSC‐derived BBB spheroids demonstrate a strong potential as a reliable BBB in‐vitro test system.

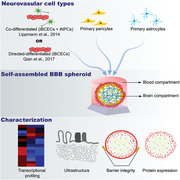

## 1 Introduction

The blood–brain barrier (BBB) is a dynamic and complex vasculature system consisting of nonfenestrated brain capillary endothelial cells (BCECs) lining the vascular lumen. The BBB in combination with pericytes (PCs) embedded within the basal lamina and astrocytes (ACs), whose end feet enclose the vasculature, forms the complex neurovascular unit (NVU).^[^ ^1^, ^2^ ^]^ This complex NVU cellular architecture and interplay allows for regulated monitoring of molecular trafficking between the blood and the brain, with the BBB posing as a major constraint in neuro‐therapeutic delivery across the brain.^[^ ^3^ ^]^ Although animal‐based models provide indispensable knowledge in drug delivery prospects into the brain, they offer limited translational abilities due to interspecies differences.^[^ ^4^ ^]^ In‐vitro models based on human induced pluripotent stem cells (hiPSCs) come to aid in abridging expensive and laborious in‐vivo drug permeability studies thereby, permitting high prospects in developing humanized in‐vitro test systems.^[^ ^5^ ^]^ These test systems can further be used for drug permeability studies,^[^ ^6^, ^7^ ^]^ viral infection studies,^[^ ^8^, ^9^ ^]^ bacterial infection studies^[^ ^10^, ^11^ ^]^ and development of fluid shear stress‐based models.^[^ ^12^, ^13^ ^]^ Recreating the BBB in‐vitro is aspiring and often limited to the use of mono‐cultured BCECs on standard cell culture inserts. To that end, 2D cell culture insert‐based models are widely used due to their versatility, high reproducibility and practicality.^[^ ^14^ ^]^ For co‐culture approaches, BCECs are seeded on the apical or “blood” side of a synthetic membrane while other NVU cell types are seeded on the basolateral or “brain” side. Such co‐culture models offer induction and maintenance of BBB properties such as enhanced barrier integrity, functionality and upregulation of BBB genes.^[^ ^6^, ^15^, ^16^ ^]^ In recent years, the use of the most suitable strategy for differentiation of hiPSCs into BCECs has been debated and discussed, with current hiPSC‐derived BCECs renamed to brain capillary endothelial‐like cells (iBCECs).^[^ ^17^, ^18^ ^]^ Due to these existing discussions around the cellular identity and maturity of iBCECs, we initiated our investigations using two differentiation strategies to derive iBCECs (Figure S1, Supporting Information). Additionally, to verify the robustness of the spheroid models and differentiation strategies, we opted to use two different hiPSC lines in spheroid generation.

Although cell culture insert‐based models are “gold standards” in BBB in‐vitro modeling, they lack direct cell‐cell and cell‐matrix interactions attributable to the semi‐permeable cell culture plastic membrane and lack of 3D cellular organizations thereby, limiting its scope in mimicking in‐vivo physiology.^[^ ^19^ ^]^ Additionally, they require a large number of cells for model establishment.^[^ ^20^ ^]^ To enable the interplay between NVU cell types, development of advanced 3D spheroid models of the BBB is in recent focus. Previous reports illustrate that cultivation of primary or immortalized NVU cells under nonadhesive conditions enables NVU cell self‐assembly resulting in the formation of multicellular spheroids with enhanced BBB functionality.^[^ ^21^, ^22^, ^23^, ^24^, ^25^ ^]^ Such models have not yet been replicated using iBCECs. Successful usage of a hiPSC‐derived BBB in‐vitro model requires it to be first characterized and benchmarked for hallmark BBB characteristics such as, gene and protein expression, morphology and intact barrier integrity accompanied with barrier permeability functions.

Here, we report for the first time the establishment and characterization of a self‐assembled 3D spheroid model of the BBB using iBCECs in combination with primary human astrocytes (ACs) and pericytes (PCs). In our studies, we compared endothelial characteristics at transcript, protein, ultrastructural and functional level, indicating differences induced by direct cellular contacts. Our results indicate maturation of iBCECs due to direct NVU contacts.

## 2 Results

### 2.1 Higher Expression of Blood–Brain Barrier Relevant Transcripts Are Observed in Spheroids Generated Using iBCECs‐Derived via Co‐Differentiation

**Figure 1:**
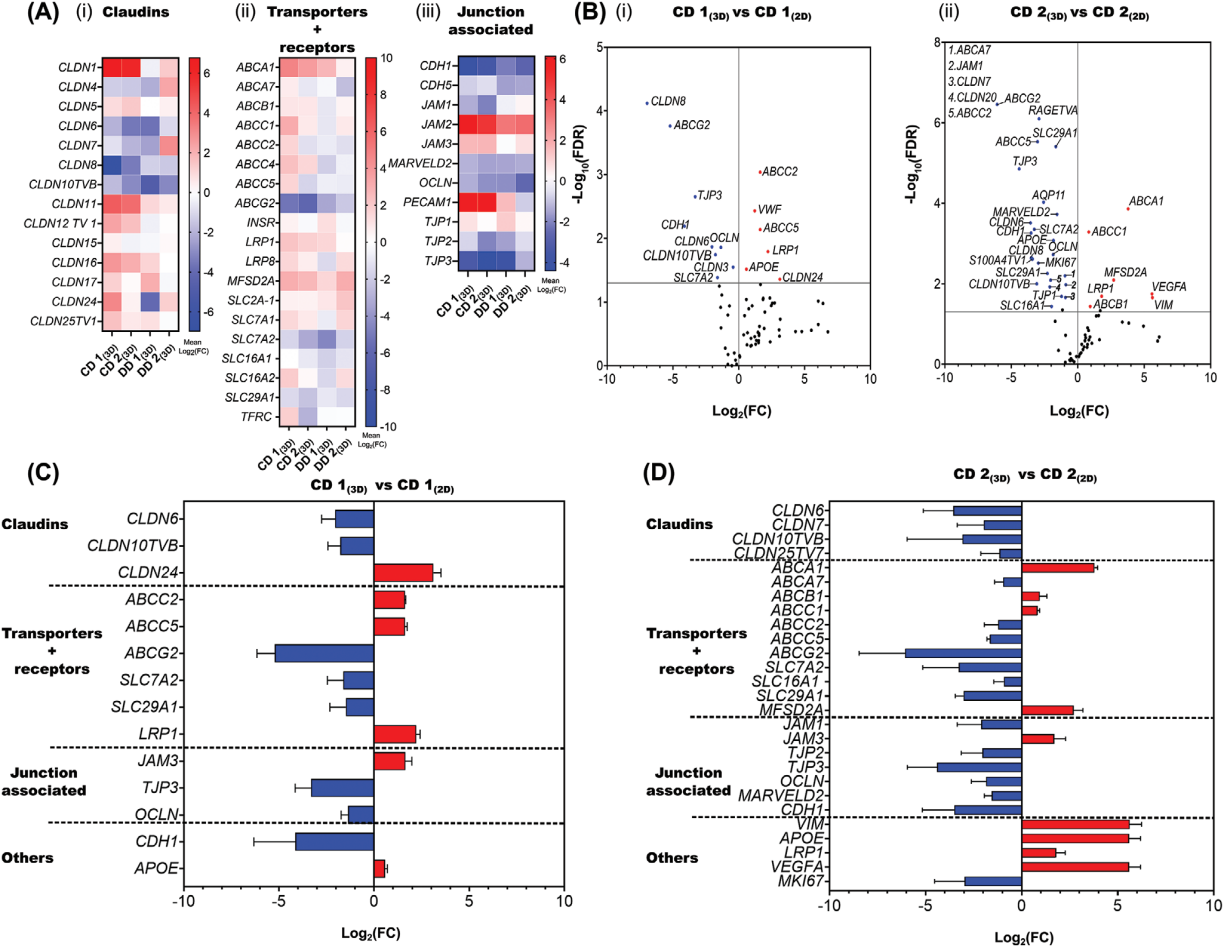
Expression of blood–brain barrier characteristic transcripts in 3D spheroids containing iBCECs in comparison to 2D mono‐cultures. Relative gene expression of relevant blood–brain barrier (BBB) transcripts in BBB spheroids were obtained via high‐throughput multiplex quantitative real time‐polymerase chain reactions. Analysis was performed for a minimum of *n* = 3 (exceptions *n* = 2 for CD 1_(3D)_, DD 1_(2D)_) biological replicates per condition using the 2^− ΔΔCt^ method with 2D mono‐culture iBCECs cultivated on cell culture inserts as a reference. Mean Log_2_ (Fold change, FC) are represented in the figure and *p* values ≤0.05 were considered as significant. Heat maps showing relevant mean Log_2_ (FC) values are represented in (A i‐iii). Volcano plots represent differentially up‐ (red) and downregulated (blue) genes. Top differentially regulated genes are labeled (B i‐ii). Significantly regulated transcripts in CD 1_(3D)_ (C), CD 2_(3D)_ (D), samples in comparison to CD 1_(2D)_ and CD 2_(2D)_ respectively are presented as bar graphs. Statistical significances were determined by paired two‐tailed t‐Test. Significantly regulated genes represent mean fold gene expression ± SD.

Using a high‐throughput multiplex quantitative polymerase chain reaction (qPCR) approach, we compared the expression of BCEC specific genes in 3D spheroids to 2D mono‐cultured iBCECs (**Figure** **1**; Figure S2, Supporting Information). Spheroids generated using iBCECs‐derived via CD showed noticeably higher number of differentially expressed genes (Figure 1B; Figure S2B and Table S1, Supporting Information). Genes associated with junction formation, transport and other targets were evaluated and displayed as heat maps of normalized Log_2_(fold change (FC)) mean values in 3D spheroids (Figure 1A). For the co‐differentiation approach (CD) we used 2 different hiPSC lines further named as CD 1 (IMR90‐4‐derived) and CD 2 (SBAD02‐01‐derived), similarly the abbreviation for directed‐differentiation is DD 1 (IMR90‐4‐derived) and DD 2 (SBAD02‐01‐derived). Mono‐cultures on cell culture inserts are denoted by subscript 2D and co‐culture spheroids with 3D. Comparing the expression of junction associated genes between the differentiation strategies showed that CD 1_(3D)_ and CD 2_(3D)_ had higher expression levels of the claudins (*CLDN*) *CLDN1*, *CLDN5*, and *CLDN11* compared to the corresponding 2D mono‐cultures. Junction adhesion molecules (*JAM*) *JAM2*, *JAM3*, *PECAM‐1* were also significantly upregulated (Figure 1A).

Epithelial cadherin (*CDH1*) and *CLDN6*, also known to be more specific to epithelial or pluripotent stem cell junctions^[^ ^26^, ^27^, ^28^ ^]^ were significantly downregulated in spheroids generated with iBCECs derived via CD strategy for both hiPSC lines (Figure 1C,D). These results collectively indicate that direct cellular contacts with ACs, PCs and iNPCs (included as mixture of cells from day 8 of differentiation are used in CD variants) induce more endothelial phenotypes in iBCECs indicating maturation of iBCECs. However, we also observed, that tight junction (TJ) markers tight junction protein 2 (*TJP2*) and occludin (*OCLN*) were significantly downregulated in spheroids generated with iBCECs‐derived via CD for both hiPSC lines. Comparison of transcript expression of transport‐associated genes between the differentiation strategies showed, that CD 1_(3D)_ and CD 2_(3D)_ exhibit similar transcript expression patterns to DD 1_(3D)_ and DD 2_(3D)_. However, hiPSC line‐based differences were observable (Figure 1C,D), ATP binding cassette subfamily C member 2 (*ABCC2*) and 5 (*ABCC5*) for example were significantly upregulated in CD 1_(3D)_ while they were significantly downregulated in CD 2_(3D)_. Importantly between the two‐hiPSC lines, the following genes were commonly regulated: ATP binding cassette subfamily G member 2 (*ABCG2*), solute carrier family 7 member 2 (*SLC7A2*), equilibrative nucleoside transporter 1 (*SLC29A1*), *CLDN6* and *CLDN10, TJP3*, *OCLN* and *CDH1* were significantly downregulated while *JAM3* and Apolipoprotein E (*APOE*) were upregulated. No such common regulations were observed within the DD samples. However, in comparison to DD 1_(2D)_ samples, DD 2_(3D)_ samples showed significant upregulation in TJ markers tight junction protein 1 (*TJP1*) and junction adhesion molecule 2 (*JAM2*) (Figure S2, Supporting Information).

### 2.2 Ultrastructural Analysis Reveals Characteristic Tight Junction Meshwork in Both Cell Culture Insert and Spheroid BBB Models Derived via Co‐Differentiation

**Figure 2:**
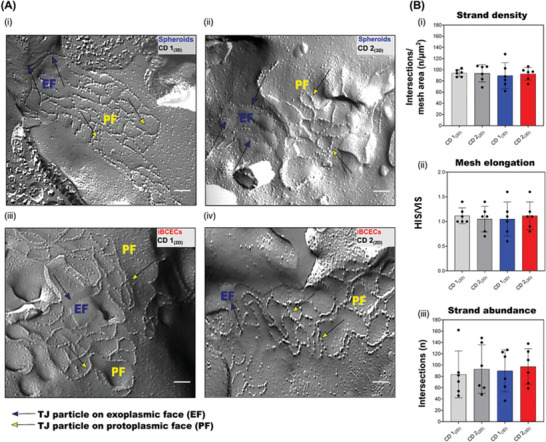
Freeze fracture electron micrographs show characteristic meshwork of tight junction strands similar to those found in brain capillary endothelial cells.

Central to the organization of barrier function at the BBB is the establishment of a complex meshwork of TJ strands composed of transmembrane TJ proteins such as *CLDN5*. These TJs seal the paracellular space between microvascular endothelial cells representing the core structure of the BBB.^[^ ^29^ ^]^ TJ strands are characterized by linearly arranged intramembranous particles. In freeze fracture electron microscopy (FFEM) images of epithelial cells, intact (and *CLDN2*‐negative) TJ strands are mainly detected as continuous (and cylindrical) fibrils on the protoplasmic fracture face (P‐face) and complementary grooves on the exoplasmic fracture face (E‐face) of the plasma membrane. In contrast, in *CLDN5‐*positive endothelial cells, TJ strands are detected as beaded particles on the E‐ and P‐face as well as grooves on the E‐face and partly ridges on the P‐face.^[^ ^30^, ^31^, ^32^ ^]^ iBCECs are reported to present a complex network of rather continuous strands at the P‐face with complementary grooves populated with spaced particles on the E‐face, fitting to expression of *CLDN1, −4* and *−5* in these cells.^[^ ^6^, ^33^, ^34^ ^]^ In the investigations reported here, TJ strands were detected as grooves containing spaced particles on the E‐face, while on the P‐face partly beaded particles and partly continuous strands were detected in both 2D as well as 3D samples of CD. Both showed similar and complex meshwork with branched strands and mixed P/E‐face associations (**Figure** **2**A). Interestingly in iBCECs derived via DD, very few TJ strands, particles or grooves on the P‐ or E‐face were identifiable with the exception of one replicate in DD 2_(2D)_ (Figure S3, Supporting Information). Due to the lack of detectable characteristic TJ strands at ultrastructural levels, quantifications were not possible with these samples. To verify differences between 2D and 3D samples of CD, and to identify, if hiPSC line‐based differences exist, we further quantified the complexity of the network using ultrastructural morphometric parameters (Figure 2B). Strand abundance (n) and strand density (n/µm^2^) are morphometric parameters that correlate with barrier function.^[^ ^35^, ^36^ ^]^ Mesh elongation is indicative of TJ strand elongation and spread uniformity. Strand density (Figure 2B‐i) presented no significant differences between the samples. CD 1_(2D)_ had strand density values of 94.2 ± 6.2 nµm^−^^2^ while CD 1_(3D)_ showed values of 89.5 ± 23.2 nµm^−^^2^ in comparison to 93.66 ± 15.3 nµm^−^^2^ for CD 2_(2D)_ and 92.76 ± 11.2 nµm^−2^ for CD 2_(3D)_. Quantification of mesh elongation (Figure 2B‐ii) also demonstrated no significant differences between the samples. CD 1_(2D)_ showed mesh elongation of 1.12 ± 0.16  while CD 1_(3D)_ had values of 1.05 ± 0.34  and CD 2_(2D)_ demonstrated values of 1.04 ± 0.27  in comparison to 1.12 ± 0.27  for CD 2_(3D)_. Strand abundance (Figure 2B‐iii) presented no significant differences between samples. CD 1_(2D)_ resulted in strand abundance values of 83.6 ± 41.5, CD 1_(3D)_ in 90.0 ± 37 while CD 2_(2D)_ resulted in strand abundance values of 93.17 ± 42.9 and CD 2_(3D)_ of 97.50 ± 31.4. No significant changes between both 2D and 3D samples derived via the CD strategy were identified by analyses of ultrastructural morphometric parameters. This implies the similarity of the TJ meshwork between both models, providing evidence of successful incorporation of iBCECs with complex sealing junctions into the spheroids. In summation, at the ultrastructural level, we were able to confirm that in cell culture insert‐based monoculture set ups as well as in spheroids generated using iBCECs derived via CD, TJs with particles on P‐ and E‐face are visible, similar to those found in human brain capillaries.^[^ ^29^ ^]^ This prompted us to investigate the barrier integrity of these TJs in BBB spheroids.

Electron micrographs were captured at 50000x magnification. Yellow arrowheads indicate tight junction (TJ) particles/fibrils on the protoplasmic face (P‐Face), blue arrowheads indicate grooves on the exoplasmic face (E‐face) that are complementary to TJ fibrils/strands on the P‐face, hence also indicative for TJ strands in the sample. The grooves are partly filled with particles. A minimum of *n* = 3 biological replicates were imaged per sample set. Scale bar = 200 nm (A, i–iv). See also Figure S3 (Supporting Information). To quantify TJ strand complexity, freeze fracture electron micrographs were overlaid with full image grids (1.9 × 1.9 µm^2^) consisting of horizontal and vertical lines, each of distance 100 nm. Quantifications were performed for a minimum of three images per sample set. Strand abundance (n) was quantified as the sum of intersections between TJ strands and overlaid grid lines, stand density as n divided by mesh area (n/µm^2^). Mesh elongation (horizontal intersecting strands (HIS)/vertical intersecting strands (VIS)) indicates the spread of the TJ network. One indicates a uniform 2D spread, two indicates 2x elongation in one direction. Measured values are represented as bar graphs with mean ± SD (B, i–iii). Ordinary one‐way ANOVA followed by Tukey's multiple comparison test was performed to identify significant differences between the samples. However, no statistically significant differences were found.

### 2.3 Blood–Brain Barrier Spheroids Possess Barrier Integrity Revealed via Electrical Impedance Spectroscopy and Small Molecule Tracer Permeability

**Figure 3:**
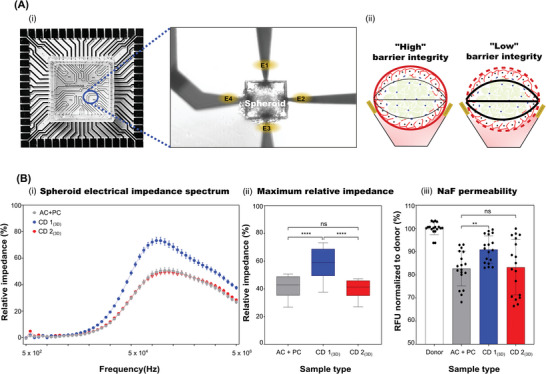
Verification of electrical impedance spectroscopy and investigation of small molecule permeability in blood–brain barrier spheroids.

Barrier integrity can be analyzed in‐vitro by monitoring transendothelial electrical resistance (TEER).^[^ ^37^ ^]^ While TEER by electrical impedance spectroscopy (EIS) based measurement on 2D cell culture inserts is well established and widely used, the analysis of 3D/spheroid cultures lacks available measurement systems. The development of micro cavity arrays (MCA; **Figure** **3**A) in combination with EIS allows the possibility for bioelectronic analysis of barrier integrity in 3D models.^[^ ^38^, ^39^, ^40^ ^]^ The chip array used to verify barrier integrity of BBB spheroids consists of several micro cavities with diameters ranging from 150 to 450 µm size (Figure 3A). For measurement purposes a micro cavity size of 300 µm (Figure 3 A, i zoomed inlet image) was used. Each measured spheroid was gently placed inside the micro cavity surrounded by four measuring electrodes (Figure 3A‐i‐ii). The complete spectra of relative impedance [%] between frequency ranges of 5 × 10^2^ and 5 × 10^6^ are depicted in (Figure 3B‐i). Maximum relative impedance [%] obtained in a frequency range of 5 × 10^4^–5 × 10^6^ is depicted in (Figure 3B‐ii). Within this selected frequency range, we were able to identify statistically significant changes within the samples. Additionally, cell line‐based differences were observed. CD 2_(3D)_ samples showed significant lower relative impedance of 41.24 ± 11% in comparison to CD 1_(3D)_ 58.28 ± 17%. AC + PC spheroids (ie. spheroids without iBCECs) also showed significant lower relative impedance of 41.72 ± 13% indicative of low barrier integrity. Although the existence of a cellular barrier in spheroids was verified, this does not substantiate to a functional barrier preventing paracellular permeation. As a first proof of concept, a permeation study using a small molecule tracer sodium fluorescein (NaF) was performed. Here relative fluorescence unit [%] (RFU%), normalized to the donor level were calculated. RFU% values correlated well with EIS measurements. Spheroids containing iBCECs showed lower permeability to NaF indicated by measurement of higher donor concentrations in the media surrounding spheroids (“apical” compartment) in comparison to AC + PC spheroids without an endothelial barrier. CD 1_(3D)_ samples showed RFU% of 91 ± 6% and CD 2_(3D)_ demonstrated RFU% of only 83 ± 12 similar to AC + PC spheroids which presented with RFU% of 83 ± 8% (Figure 3B‐iii), corroborating EIS measurements. In conclusion, we were able to demonstrate, that barrier integrity of BBB spheroids can be quantified and validated by EIS measurement and studying small molecule tracer permeability.

An impedance chip array with a cavity size of 300 µm was used to determine barrier integrity in blood–brain barrier (BBB) spheroids. Each spheroid was placed into a cavity, such that four measuring electrodes surrounded it (A, i). Spheroids which contain endothelial cells represent high barrier integrity (red lines), black lines indicate electrical field lines (A, ii). A minimum of 3 independent biological replicates with 15 spheroids per replicate (ie., *n* = 45 spheroids) were measured and calculated relative impedance values are represented in percentage as mean ± SD for each condition (B, i‐ii). Relative impedance [%] as mean ± SEM for a frequency range of 5 × 10^2^–5 × 10^6^ is represented in (B, i). Relative impedance [%] as mean ± SD corresponding to values extracted from frequency range of 5 × 10^4^–5 × 10^6^ is represented in (B, ii). Small molecule permeability of 10 µm sodium fluorescein (NaF) is represented in terms of mean relative fluorescence unit (RFU%) normalized to donor samples ± SD, (B, iii). Statistical significances were determined by one‐way ANOVA, and multiple comparisons of mean values were assessed by the Tukey´s multiple comparison test (B, ii) or Dunnett´s multiple comparison (B, iii). ^****^ indicates *p* < 0.0001, ^***^ indicates *p* ≤ 0.001, ns indicates no significances.

### 2.4 Blood–Brain Barrier Spheroids Possess Tissue Specific Protein Expression

**Figure 4:**
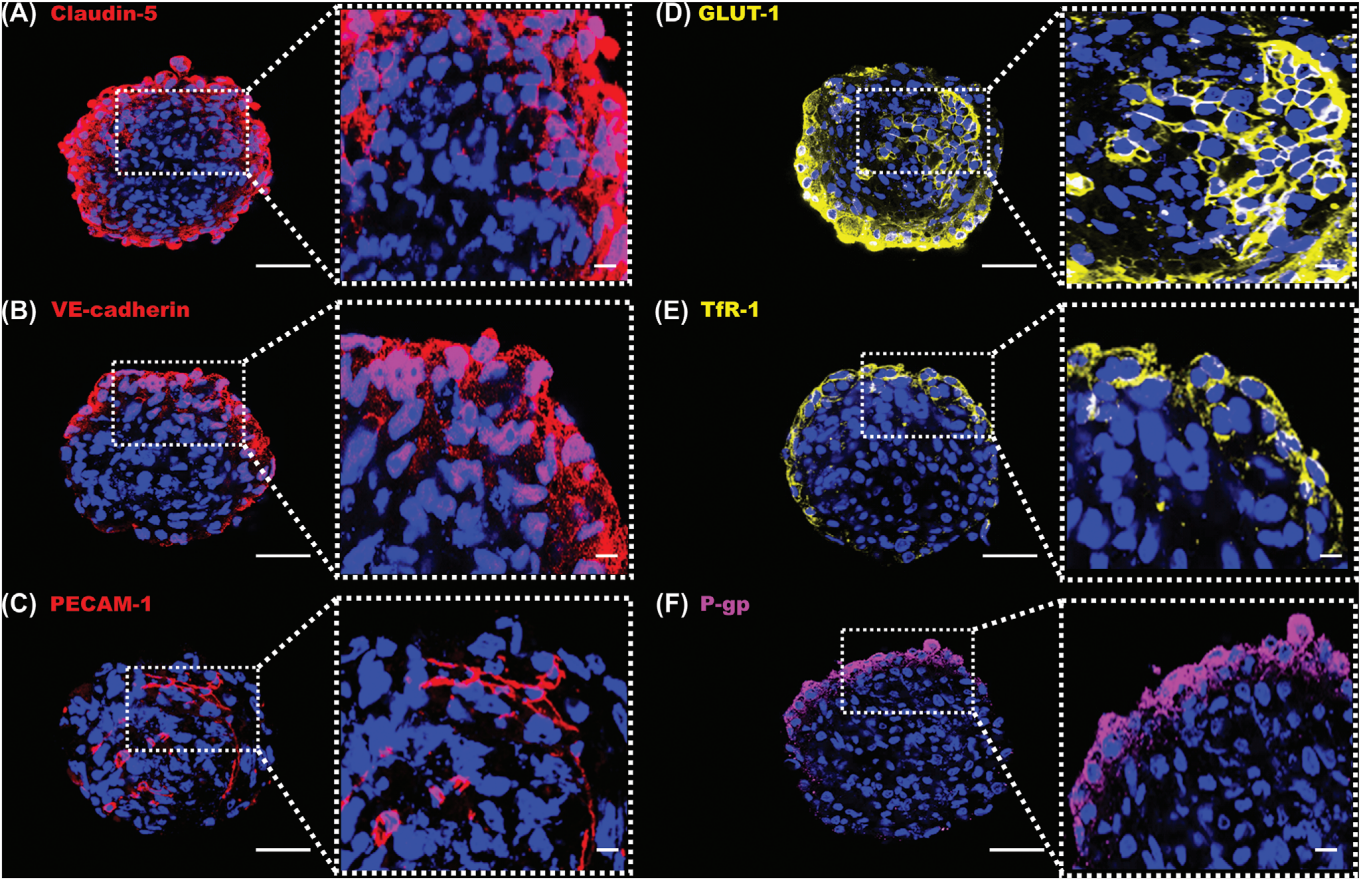
Verification of tissue specific protein expression.

Functionality of iBCECs and paracellular permeability is linked to the expression of junctional molecules and relevant transporters/receptors. Therefore, verification of key junctional molecules such as claudin‐5, vascular endothelial cadherin (VE‐cadherin), platelet endothelial cell adhesion molecule (PECAM‐1) and expression of common transporters/receptors such as glucose transporter‐1(GLUT‐1), P‐glycoprotein (P‐gp), and transferrin receptor‐1 (TfR‐1) in BBB spheroids is critical. With the aid of confocal laser scanning microscopy (CLSM) key BCEC‐specific markers were identified in spheroids with hiPSC line‐specific differences. Strong expression of claudin‐5 was observed in the border area of the spheroids, revealing accumulation of BCECs as outer spheroid layer at higher magnifications (**Figure** **4**A; Figure S4, Supporting Information). VE‐cadherin was observed to stain the nucleus and the cytoplasm rather than defined staining to cellular borders in 3D as well as 2D samples (Figure 4B; Figure S4, Supporting Information). Surprisingly, there were hiPSC line‐based differences in expression of PECAM‐1. Hardly any PECAM‐1 expression was identifiable in CD 2_(3D)_ samples (Figure S4C, Supporting Information) while CD 1_(3D)_ samples showed a few cells positive for PECAM‐1 with distinctive elongated staining patterns (Figure 4C). Transporters such as GLUT‐1, TfR‐1 and P‐gp were expressed similarly in both CD 1_(3D)_ and CD 2_(3D)_ samples. GLUT‐1 was homogenously expressed in cellular membranes extending as both a ring around the spheroid and toward the inside of each spheroid (Figure 4D; Figure S4, Supporting Information). TfR‐1 and P‐gp were expressed only as a ring on the outer surface of each spheroid, mainly staining cellular borders (Figure 4E,F; Figure S4, Supporting Information). Additional to expressed characteristic BCEC markers, incorporated ACs, PCs as well as iNPCS (in case of CD‐differentiated cells) were identified via immunohistochemistry (Figure S1, Supporting Information). ACs stained positively for glial fibrillary acidic protein (GFAP) and were expressed throughout the core of the spheroid (Figure S1i‐ii, Supporting Information). GFAP, a principal AC marker was observed as short, thick and branching protrusions. PCs showing positive expression for platelet‐derived growth factor receptor‐beta (PDGFR‐β) were localized as a ring around the spheroids and not in the core (Figure S1iii‐iv, Supporting Information). iNPCs stained positively for nestin (Figure S1v‐vi, Supporting Information) and SRY‐box transcription factor‐1 (SOX‐1) (Figure S1vii‐viii, Supporting Information). SOX‐1, the earliest and most specific known marker for mammalian iNPCs was located in cellular nuclei of BBB spheroids. Nestin, an intermediate filament protein was expressed as long filamentous protrusions. Both SOX‐1 and nestin positive cells were found at spheroid cores together with ACs. Importantly we identify that iBCEC‐based BBB spheroids cannot be cultured for prolonged durations as they lose their compactness and structural integrity (Figure S1B, Supporting Information).

CD 1_(3D)_ samples were investigated for the expression of key BCEC markers in *n* = 3 biological replicates. Images were captured at 40 × magnification through a maximum Z stack capacity of the confocal laser scanning microscope and representative slice images of depth ranging from ≈50 to 70 µm are presented via single Z stacks (scale bar = 50 µm). Zoomed in areas are indicated on the right panel of each spheroid (scale bar = 20 µm). Nuclei (blue) were labeled with Sytox Red dead cell stain. Expression and localization of junctional proteins claudin‐5 (A), vascular endothelial‐cadherin (B) and platelet endothelial cell adhesion molecule‐1 (C). Expression of transporter/receptor markers glucose transporter‐1 (D), transferrin receptor‐1 (E) and P‐glycoprotein (F) were verified in blood–brain barrier (BBB) spheroids. See also Figures S1 and S4 (Supporting Information).

## 3 Discussion

Recent reports indicate that iBCECs are not entirely analogous to in‐vivo BCECs and lack accepted generic endothelial profiles.^[^ ^18^, ^41^ ^]^ One of the major reasons could be lack of complete cellular maturation, known to be predominantly true for several hiPSC‐derived cell types as in‐vitro differentiations often leads to achievement of a target cell type that is not completely analogues to in‐vivo counterparts.^[^ ^42^ ^]^ HiPSC‐derived cell types are often immature and fetal‐like based on transcriptional analyses. To achieve maturation, addition of complex supplements, overexpression of relevant target genes or co‐culture with essential cell types are required.^[^ ^43^ ^]^ With regard to the BBB, establishment of direct NVU cellular contacts is possible in 3D spheroid models. This model has been previously developed and investigated using immortalized or primary cells,^[^ ^21^, ^22^, ^25^ ^]^ but never with hiPSC‐based setups. During in‐vivo development, cues from the entire NVU are required for proper maturation of the BBB. Cues that are unavailable in mono‐culture iBCEC conditions can be bridged with BBB spheroids. In order to identify an optimal iBCEC cell source that can provide most common BBB attributes, we generated and compared BBB spheroids from two widely reported iBCEC differentiation strategies using two hiPSC lines. Our results indicate that the suitable choice of iBCECs to be used in BBB spheroid generation would be derived via CD.

Direct contact with NVU cell types leads to maturation of iBCECs, especially visible by downregulation of stem cell‐like *CLDN6* and epithelial *CLDN4, −7, −8, −10TVB* ^[^ ^44^ ^]^ as well as *CDH1* transcript levels and upregulation of BCEC‐specific transcripts such as *PECAM‐1*. The biological significance of the detected upregulation of *CLDN1, −16, −24, and −25* and *CLDND1* is not fully clear. For the *CLDN24/−25*‐related mouse *CLDN21*, formation of paracellular cation channels was suggested^[^ ^45^ ^]^ and channels potentially formed by the related *CLDN24/−25* would not fit to low ion conductance of iBCECs^[^ ^6^ ^]^ (Figure 3). *CLDND1* was reported to be expressed in BCECs and suggested to contribute to BBB TJs, however, the data was ambiguous.^[^ ^46^ ^]^

The characteristic ultrastructural TJ meshwork formed by iBCECs in BBB spheroids showed high complexity (many strands and branches) as well as comparable strand and mesh structures to mono‐culture iBCECs. Previous reports show that iBCECs in combination with NVU cell types on cell culture inserts show a tendency for higher TJ complexity compared to mono‐cultures respectively.^[^ ^6^ ^]^ In this work, BBB spheroids showed a slight tendency for increase in TJ strand abundances, corroborating the positive effects of NVU cell types on iBCECs.

In most epithelial cell types, TJ strands consist of multiple claudin subtypes and their barrier/permeability properties depend on claudin combinations.^[^ ^36^, ^44^ ^]^ In contrast, BCEC TJs are dominated by *CLDN5*.^[^ ^47^, ^48^ ^]^ However, to which extent other claudins are present is controversial. Some studies indicate only a minor role of additional claudins^[^ ^48^, ^49^ ^]^ while others suggest a functionally relevant role of one or several of the following claudins: *CLDN1, −3, −4, −6, −11, −12, −D1*.^[^ ^50^, ^51^, ^52^, ^53^ ^]^ These differences are likely to be due to the different cell sources for model development and analytical methods used in the respective studies.

The tight barrier properties of BBB TJs correlate with the mixed presence of strand particles on the E‐ and the P‐face, with P‐/E‐face partition ratio of up to 55% associated with lower solute permeability than lower ratios.^[^ ^54^ ^]^ Co‐expression of *CLDN5* with other claudins such as *CLDN3* or *CLDN1* increases the P‐/E‐face ratio.^[^ ^52^, ^53^, ^55^, ^56^ ^]^ In this study, the iBCECs, monolayers as well as spheroids clearly showed intramembranous TJ strand particle on the P‐ and the E‐face. This together with the mRNA expression data (Figure 1) is indicative for the presence in TJ strands of *CLDN5* and/or *CLDN6* (E‐face) and one or several claudins out of *CLDN1, −4, −7, −10B, −11, −15 (P‐face)*.^[^ ^33^, ^34^, ^57^ ^]^ However, *CLDN11*‐containing strands are rather unbranched and parallel,^[^ ^33^, ^46^ ^]^ in contrast to the meshes of branched strands detected here, and *CLDN10* and *−15* form cation channels not fitting to low ion permeability in iBCEC monolayers and spheroids indicated by high TEER^[^ ^6^ ^]^ and EIS (Figure 3), respectively. In sum, the freeze fracture analysis demonstrated that CD‐derived spheroids contain barrier‐forming TJ strands with at least partly brain‐endothelial features, and it supports the idea that in endothelial TJs, *CLDN5* can be supported by other claudins such as *CLDN1, −4, −6, −7*. Together with the mRNA expression data (Figure 1), these hints suggest that *CLDN5* and *−1* are the strongest contributors to TJ formation in the spheroids.

Quantification of TJ integrity, paracellular permeability and transporter functionality of 2D in‐vitro BBB models is performed via various techniques and methods such as EIS measurements and quantitative measurement of tracer fluxes. In order to assess the barrier integrity of BBB spheroids, relative impedance was measured in CD 1_(3D)_, CD 2_(3D)_ and AC + PC spheroids. Statistically significant changes were observed within the samples, along with hiPSC line‐based differences. This was collated with a small molecule tracer flux study indicating overall higher donor concentrations in the medium surrounding spheroids, which contained iBCECs, thereby indicative of low penetration into the spheroid core. As these results are only comparisons relative to AC + PC spheroids, they are not comparable to other barrier integrity methods used in BBB literature. Therefore, in order to assess the full potential of the generated model, further studies such as assessment of permeability levels of anti‐TfR antibodies, cyclic peptides, active transport of substances, and evaluation of efflux pump activities must be carried out to identify qualitative discriminatory ability of BBB spheroids.

Importantly, we observed hiPSC line‐specific differences in EIS measurements and in protein expression of PECAM‐1. One possible explanation for this could be the epigenetic memory biases of hiPSCs in differentiation and maturation to iBCECs.

In order to account for hiPSC‐line based differences, we suggest that initial characterization of key markers should be conducted in BBB spheroids when using other hiPSC lines. Biases originating from methylation signatures and histone modifications of hiPSC tissue origin drive phenotypical differences and donor‐dependent variations that affect differentiation potentials and functional properties.^[^ ^58^, ^59^ ^]^ This highlights the need for the usage of “gold standard” hiPSC lines^[^ ^60^ ^]^ in BBB model development to maintain reproducibility.

To conclude, we introduce a novel iBCEC‐based 3D BBB spheroid fulfilling first fundamental characterization criteria in BBB in‐vitro modelling. We highlight, that initial characterizations are important in the application of BBB spheroids, as there can be hiPSC line and differentiation strategy‐based contrasts. The next steps regarding deciphering functional aspects of the BBB spheroids require investigation into the permeability and transport of reference substances and biologicals, characterizing the functionality of active transporters and the polarization of the cells. Benchmarking in‐vivo partition coefficients will be key in proving spheroid applicability.^[^ ^61^ ^]^ Some novel and necessary options would be to assess pharmacokinetic and pharmacodynamic parameters via classically used methods.^[^ ^62^ ^]^ Another strategy would be to determine intra spheroidal concentrations of antibodies, peptides and drug substances via enzyme‐linked immunosorbent assays or liquid chromatography mass spectrometry. The next steps would include addressing the standardized production of spheroids with high reproducibility in medium‐ to high‐throughput formats by using robotic systems. It would further be important to assess how the spheroids can be applied as an in‐vitro screening platform for brain‐penetrating molecules. Two other unexplored applications of BBB spheroids will be the investigation of mechanisms involving pathogenic invasions of the BBB and identification of specific extracellular matrix components and interactions that allows for self‐assembly. Identification of possibilities for spheroids to be cryopreserved would allow the opportunity of providing ready to use kit formats for substance testing, this would prove especially useful when generating isogenic BBB spheroids.

## 4 Limitations of the Study

Although BBB spheroids are scalable, cheaper and simpler to fabricate, requiring few reagents and cell numbers per spheroid, their formation is more sophisticated than putting together a solution with various cell types. Specific interactions between each NVU cell type and organization into localized structures determine how each cell works together in the formed spheroid. Since no scaffolds or extrinsic parameters are used, the localization of each cell is primarily driven by cell–cell interactions and the path to fully understand the application potential of this model is still in its infancy. However, the possibilities are immense. It should be noted that due to limited prolonged culture possibilities, applications requiring long‐term monitoring cannot be conducted using BBB spheroids in this stage of research.

Although we were able to show positive barrier integrity in spheroids, we could not confirm that each spheroid is completely covered by an iBCEC monolayer.

It has been reported that CLSM is not the most appropriate technique to monitor the inner cellular structures of BBB spheroids as it only allows a Z‐stack scanning depth of 100 µm.^[^ ^63^ ^]^ We faced similar difficulties in whole mount imaging; thereby we speculate that the visualization of whole spheroids can be possible in the future by firstly employing tissue‐clearing strategies and imaging via light sheet microscopy.

We additionally demonstrated low small molecule permeability in spheroids; however, we could not correlate these results to standard barrier integrity and BBB flux measurements in BBB literature. This has to be conducted using comparable measurement devices and methods. These studies will be indispensable to prove and enhance the value of BBB spheroids. Furthermore, the specific influence of the different cellular subtypes of the NVU on the transcriptome of iBCECs within the specific 3D microenvironment of the BBB spheroids should be investigated in detail in future studies. Further limitations in using BBB spheroids could be handling of large imaging data files and downstream analysis techniques that are not easily accessible to all laboratories.

## 5 Experimental Section

### Preparation of Cell Culture Coatings—Matrigel Coating for Cultivation of iBCECs

For optimal adherence of iBCECs, 24‐well cell culture inserts were coated with 200 µg mL^−1^ of Matrigel. 0.5 mg Matrigel was mixed with 5 mL cold DMEM/F‐12 minus L‐Glutamine. The prepared coating solution was distributed as 100 µL per 24 well cell culture insert followed by incubation for 1 hour (h) at room temperature (RT). Post incubation, the cell culture inserts were used directly for cell seeding.

### Preparation of Cell Culture Coatings—Matrigel Coating for Cultivation of hiPSCs

For optimal adherence of hiPSCs, 6‐well Nunclon delta surface plates were coated with 100 µg mL^−1^ of Matrigel. Matrigel (0.5 mg) was mixed with 12 mL cold DMEM/F‐12 minus L‐Glutamine. The prepared coating solution was then distributed as 1 mL per well followed by incubation for 1 h at RT. Post incubation, the plates were either used immediately or topped with another 1 mL per well DMEM/F‐12 minus L‐Glutamine, in order to prevent drying of the coating. The plates were stored at 4 °C for a maximum of 7 days after enwrapping the sides with Parafilm.

### Preparation of Cell Culture Coatings—Poly‐L‐Lysine‐Coating for ACs and PCs Cultivation

For optimal adherence of primary human ACs and PCs, T‐75 cell culture flasks were coated with 10 mL of 10 µg mL^−1^ poly‐L‐Lysine (PLL). The cell culture flask was then incubated for a minimum of 1 h at 37 °C. The flasks were either used directly or stored at 4 °C for a maximum of 7 days after enwrapping the cap of the flask with Parafilm.

### Cell Culture Specifications—Freezing, Thawing and Passaging of Primary ACs and PCs

Upon reaching a confluency of 80–90%, ACs or PCs were detached via incubation at 37 °C for 10 min (min) with 7 mL Accutase per T‐75 cell culture flask. Detached cells were collected and centrifuged for 5 min at 270 x g, followed by dissolution of the cell pellet in cell specific pre‐cooled freezing medium (80% cell specific medium + 10% fetal bovine serum (FBS) + 10% DMSO). 1 × 10^6^ mL^−1^ were transferred into a cryovial and stored for 24 h at −80 °C in a Mr. Frosty freezing aid to guarantee a temperature decrease of 1 K min^−1^, before further cryopreservation in liquid nitrogen. Revival of cryopreserved cells was performed via resuspension of each cryovial in 10 mL of respective medium. Cells were plated at 1 × 10^6^ cells per T‐75 cell culture flask for further propagation. Medium was refreshed in a 3‐days rhythm. Passaging of the cells was also performed at a confluency of 80–90 %. For passaging, both cell types were treated as mentioned above and seeded at a density of 1 × 10^6^–2 × 10^6^ per T‐75 flask for ACs or 0.5 × 10^6^–1 × 10^6^ per T‐75 flask for PCs. Cells up to passage 7 were used for all experiments.

### Cell Culture Specifications—Freezing and Thawing of hiPSCs

Upon reaching a confluency of 70 – 80%, hiPSCs were detached as colonies using 0.5 mL of respective enzymes. Gentle Cell Dissociation Reagent (GCDR) was used for IMR90‐4 hiPSCs and Versene solution was used for SBAD‐02‐01 hiPSCs. The colonies were incubated with respective enzymes for 1–2 min at 37 °C. hiPSC colonies were gently collected using a 1000 µl pipette and centrifuged. The pellet was carefully resuspended in precooled freezing medium (90% KnockOut Serum replacement + 10% DMSO) such that the colonies were left intact. Colonies were collected such that each cryovial contained cells from one well of a 6 well. The cryovials were then stored for 24 h at −80 °C in a Mr. Frosty freezing aid to guarantee a temperature decrease of 1 K min^−1^, before further cryopreservation in liquid nitrogen. Revival of cryopreserved cells was performed via resuspension of the thawed cell suspension of each cryovial in 9 mL mTeSR^1^ + 10 µm Y27632 followed by centrifugation. The pellet was then further resuspended in 4 mL mTeSR^1^ + 10 µm Y27632 and redistributed into two wells each of a precoated 6 well plate.

### Cell Culture Specifications—Passaging and Maintenance of hiPSC line IMR90‐4

Upon reaching a confluency of 50–80%, hiPSC line IMR90‐4 was split for maintenance cultures. In detail, the cell culture medium was aspirated, followed by incubation with 1 mL GCDR per 6 well at 37 °C for 2 min. As soon as the colony edges started to detach, the GCDR solution was aspirated carefully and the colonies were washed once with 1 mL PBS^−^ per well. PBS^−^ was then aspirated and 1 mL of mTeSR^1^ + 10 µm Y27632 was added per well. The colonies were then completely detached from the cell culture surface using a cell scraper. Per mL of medium no more than two resuspension steps were performed in order to detach the colonies into smaller fragments. The colonies were then seeded in a ratio of 1:10 to 1:20 on Matrigel coated 6‐well plates in a total of 2 mL per well mTeSR^1^ + 10 µm Y27632. For the first 24 h of culture 10 µM Y27632 was added as an apoptosis inhibitor. Daily medium change was then performed with 2 mL per well of mTeSR^1^.

### Cell Culture Specifications—Passaging and Maintenance of hiPSC line SBAD‐02‐01

Upon reaching a confluency of 50–80%, hiPSC line SBAD‐02‐01 was split for maintenance cultures. In detail, the cell culture medium was aspirated, followed by incubation with 1 mL Versene solution per 6 well at 37 °C for 1 min. As soon as the colony edges started to detach, Versene solution was aspirated carefully and the colonies were washed once with 1 mL PBS^−^ per well. PBS^−^ was then aspirated and 1 mL of mTeSR^1^ + 10 µm Y27632 was added per well. The colonies were then completely detached from the cell culture surface by gentle pipetting. Per mL of medium no more than two resuspension steps were performed in order to detach the colonies into smaller fragments. The colonies were then seeded in a ratio of 1:4 to 1:6 on Matrigel coated 6‐well plates in a total of 2 mL per well mTeSR^1^ + 10 µm Y27632. For the first 24 h of culture 10 µM Y27632 was added as an apoptosis inhibitor. Daily medium change was then performed with 2 mL per well of mTeSR^1^.

### Cell Culture Specifications—Derivation of iBCECs via Co‐Differentiation (CD)

CD of hiPSCs into iBCECs and iNPCs was performed as previously reported.^[^ ^64^ ^]^ In detail, at d‐3, hiPSCs were detached via incubation with 1 mL per well Accutase for 7 min at 37 °C in order to obtain single cells. Isolated single cells were centrifuged for 5 min at 270 xg, counted and then seeded at a density of 7.5 × 10^3^ cells cm^−2^ onto Matrigel coated 6‐well plates. Seeding was performed in 2 mL per well mTeSR^1^ including 10 µM Y27632 for the first 24 h. From the following day, medium was changed daily and hiPSCs were proliferated in 2 mL per well mTeSR^1^ without Y27632. hiPSC density was monitored by counting one representative well in order to determine the optimal starting point of differentiation. Cells from the respresentative well were detached using 0.5 mL Accutase with incubation for 7 min at 37 °C. If the hiPSCs reached an optimal cell density of 2.5–3.5 × 10^4^ cells cm^−2^, CD to iBCECs and iNPCs was initiated by switching the medium to unconditioned medium (UM) (DMEM‐F12 minus L‐Glutamine 78.5%, Knock out serum replacement 20%, MEM‐Non Essential Amino Acids Solution 1%, 1 mM L‐Glutamine 1%, 0.1 mm β‐Mercaptoethanol (1:500)) for 6 days with daily media changes. For iBCEC specification the medium was changed to 4 mL per well EC++ medium (Human endothelial serum free medium 100%, B27 (1:200), human basic fibroblast growth factor 20 ng mL^−1^, 10 µm retinoic acid) at d6. On d7 no medium change was performed. On d8 of differentiation, cells were treated with 2 mL per well Accutase with incubation for 30 min at 37 °C. Cells were then collected, centrifuged for 5 min at 270 xg and resuspended in EC++ medium. Counted cells were then purified by sub‐passaging at a density of 1 × 10^6^ cells cm^−2^ in EC++ medium onto 200 µg mL^−1^ Matrigel coated 24 well cell culture inserts. Cells on top of the insert membrane were seeded in a total volume of 200 µL cell suspension with an additional 850 µL EC++ medium in the basolateral compartment. Medium was changed to 200 µL EC+ medium (Human endothelial serum free medium 100%, B27 (1:200)) in the apical compartment and 850 µL EC+ medium in the basolateral compartment on d9. On d10 purified iBCECs were used for downstream 2D applications. Since two different hiPSc lines were used for differentiations, 2D cultures of iBCECs derived via CD are further labeled as CD 1_(2D)_ for IMR90‐4‐derived cells and CD 2_(2D)_ for SBAD‐02‐01‐derived cells.

### Cell Culture Specifications—Derivation of iBCECs via directed differentiation (DD)

DD of hiPSCs into iBCECs was performed as previously reported.^[^ ^65^ ^]^ In detail, at d‐3, hiPSCs were detached by incubation with 1 mL per well Accutase per well for 7 min at 37 °C to obtain single cells. Isolated single cells were centrifuged for 5 min at 270 xg, counted and then seeded at a density of 3.5 × 10^3^ cells cm^−2^ onto Matrigel coated 6‐well plates. Seeding was performed in 2 mL per well mTeSR ^1^ including 10 µm Y27632 for the first 24 h. From the following day, medium was changed daily and hiPSCs were proliferated in 2 mL per well mTeSR^1^ without Y27632. Once the colonies reached a confluency of ≈70–80%, differentiation process was initiated. At d0 the medium was changed to DeSR1 (DMEM‐F12 minus L‐Glutamine 100%, MEM‐Non Essential Amino Acids Solution (1:100), GlutaMAX Supplement (1:200), 0.1 mM β‐Mercaptoethanol (1:500), 6 µM CHIR99021). On d1, the medium was switched to DeSR2 (DMEM‐F12 minus L‐Glutamine 100%, MEM‐Non Essential Amino Acids Solution (1:100), GlutaMAX Supplement (1:200), 0.1 mm β‐Mercaptoethanol (1:500), B27 (1:50)) for 5 days with daily medium changes. On d6 medium was changed to 4 mL per well hECSR1 (Human endothelial serum free medium 100%, B27 (1:50), human basic fibroblast growth factor 20 ng mL^−1^, 10 µm retinoic acid) to initiate iBCEC specification. No medium change was performed on d7. On d8 of differentiation, the cells were treated with 2 mL/well Accutase with incubation for 30 min at 37 °C. The cells were then collected and resuspended to yield single cells. Counted cells were then purified by sub passaging at a density of 1 × 10^6 ^cells cm^−2^ in hECSR1 medium onto 200 µg mL^−1^ Matrigel coated cell culture surfaces. In 24 well transwell inserts, cells on top of the insert membrane were seeded in a total volume of 200 µL cell suspension with an additional 850 µL hECSR1 medium in the basolateral compartment. On d9 medium was changed to hECSR2 ((Human endothelial serum free medium 100%, B27 (1:50)), with 200 µL medium in the apical compartment and 850 µL medium in the basolateral compartment. On d10 purified iBCECs were used for downstream applications. Since two different hiPSC lines were used for differentiations, 2D cultures of iBCECs derived via DD are further labeled as DD 1_(2D)_ for IMR90‐4‐derived cells and DD 2_(2D)_ for SBAD‐02‐01‐derived cells.

### Cell Culture Specifications—Generation of BBB Spheroids

AggreWell 800 microwell culture plates were used in order to prepare spheroids. The plates were pre‐prepared by coating with Anti‐Adherence Rinsing Solution in order to prevent cell attachment. Each well of the microwell plate was coated with 500 µL of Anti‐Adherence Rinsing Solution, followed by centrifugation for 5 min at 270 xg. Post centrifugation, the wells were visualized microscopically to ensure that all micro‐cavities were free from bubbles. In case of bubble formation, the centrifugation steps were repeated. Anti‐Adherence Rinsing Solution was then aspirated and the wells were rinsed once with 500 µL DMEM/F12 minus L‐Glutamine per well. When pooled cell mixtures were ready for seeding, the medium was aspirated and respective pooled cell suspensions (compare **Table** **1**) were added into each well in a total of 2 mL spheroid media. The plate was then centrifuged at 300 g for 5 min. Post centrifugation all wells were visualized microscopically to ensure that the cells filled the microcavities. The plate was then placed into the incubator at 37 °C and 5% CO_2_ for 48 h before usage of spheroids for further analysis. Cellular numbers used for each spheroid condition and media specifications are mentioned in Tables 1 and **2**. Since two different hiPSC lines were used for differentiations, 3D cultures of iBCECs derived via CD were further labeled as CD 1_(3D)_ for IMR90‐4‐derived cells and CD 2_(3D)_ for SBAD‐02‐01‐derived cells. 3D cultures of iBCECs derived via DD are further labeled as DD 1_(3D)_ for IMR90‐4‐derived cells and DD 2_(3D)_ for SBAD‐02‐01‐derived cells. For CD 1_(3D)_ and CD 2_(3D)_ samples cell mixtures from d8 were used in order to incooperate both iBCECs and iNPCs into the spheroids. For DD 1_(3D)_ and DD 2_(3D)_ iBCECs from d10 were used in preparing spheroids. For prolonged culture, medium was changed on the spheroids every alternate day.

**Table 1:** Cell numbers and media used in spheroid generation.

SPHEROID TYPE	ACs	PCs	d8 iBCECs + iNPCs	d10 iBCECs
AC+PC	300 000	300 000	x	x
CD_(3D)_	300 000	300 000	600 000	x
DD_(3D)_	300 000	300 000	x	300 000

**Table 2:** Spheroid medium composition.

Components	Amount
hESFM	48%
AC medium	25%
PC medium	25%
B27	1:200
hbFGF, 100 µg mL^−1^	20 ng mL^−1^
Human serum	2%
Retinoic acid, 10 mm	10 µm
Wnt‐7a, 10 µg mL^−1^	10 ng mL^−1^

### Other Methodologies—Ribonucleic Acid (RNA) Sampling and Extraction

RNA extraction was performed using the RNeasy Micro Kit as per kit instructions. Samples were collected from a minimum of three 24 well cell culture inserts per iBCEC mono‐culture or from one complete AggreWell consisting of 300 spheroids per biological replicate. iBCEC mono‐culture samples were gently cut using a scalpel and placed into a 1.5 mL Eppendorf tube, 350 µL of lysis buffer was added into the tube and the samples were immediately frozen at −80 °C until extraction. For the spheroids, samples were collected from one complete AggreWell into a 15 mL falcon tube. Remaining media was removed gently using a 1000 µL pipette, 350 µL of lysis buffer was then added and the samples were passed through a 2 mm gauge needle several times in order to homogenize the spheroids, additionally samples were passed through a QIAshredder homogenizer mini spin column. Post homogenization, samples were stored in 1.5 mL eppis at −80 °C until extraction. Only RNA which fulfilled the purity ratio (A260/A280), falling in the range of 1.8–2 was used for downstream applications.

### Other Methodologies—Complementary Deoxyribonueclic Acid (cDNA) Synthesis

cDNA synthesis was carried out using the High‐Capacity cDNA Reverse Transcription Kit from Applied Biosystems. To carry out chip‐based quantitative real‐time polymerase chain reaction (qPCR) analysis, a minimum amount of 250 ng µL^−1^ RNA was used. It was assumed that 1 µg of cDNA was obtained from 1 µg of RNA, therefore, the corresponding sample amount was calculated from isolated RNA concentrations and transferred to a 0.5 mL Eppendorf tube. As per the kit instructions, a total volume of 20 µL of cDNA was synthesized. Component mixtures are specified in (**Table** **3**) and details of thermal cycler run is specified in (**Table** **4**). Generated cDNA was then stored at −25 °C.

**Table 3:** Mixture components for cDNA synthesis.

Component	Volume [µL]
10x reverse transription buffer	2,0
25x Deoxyribonucleotide TriPhosphate mix (100 mm)	0,8
10x reverse transription random primers	2,0
MultiScribe Reverse Transcriptase	1,0
RNase Inhibitor	1,0
Nuclease free water	x
250 ng µL^−1^ RNA sample	x
Total volume per reaction	20

**Table 4:** Run settings for cDNA synthesis.

Step	Temperature [°C]	Time [min]	Cycle
Priming	25	10	1x
Reverse transcription	37	120	1x
Inactivation	85	5	1x
Hold	4	∞	1x

### Other Methodologies—High‐Throughput Multiplex Quantitative Polymerase Chain Reaction (qPCR) 0 of Relevant BBB Transcripts

For high‐throughput multiplex qPCR of relevant BBB transcripts, 250 ng RNA per sample was transcribed into a final volume of 20 µL cDNA. For pre‐amplification of the samples Qiagen Mastermix and HotStar PlusTaq Polymerase combined with the tenfold concentration of gene targeting primers was used.^[^ ^66^ ^]^ High‐throughput qPCR was performed using the BiomarkSystem (Fluidigm) including an IFC Controller HX and 96.96 Dynamic ArraysIFC with the run settings described in (**Table** **5**) was used for multiplexing.

**Table 5:** Run settings used for high‐throughput multiplex qPCR.

Step	Temperature [°C]	Time	Cycle
Initial activation	95	15 min	1
Denaturation	95	40 s	18
Annealing	60	40 s	18
Annealing	80	40 s	18
Annealing	72	40 s	18
Final extention	72	7 min	1

### Other Methodologies—Freeze Fracture Electron Microscopy

A minimum of 100 spheroids or three cell culture inserts per condition were first washed with 1 mL PBS^+^. Following this 1 mL of 2.5%, glutaraldehyde solution was added to the samples as a fixative for 2 h at RT. The samples were washed again with PBS^+^ and stored in 0.025% glutaraldehyde at RT until sample processing.^6^ For analysis, FFEM images captured at 50 000× were quantified for TJ complexity. FFEM images were overlaid with full image grids of size 1.9 µm2 × 1.9 µm^2^ with horizontal and vertical lines, each100 nm apart. TJs were redrawn using a pencil tool in ImageJ for easier identification. Quantifications were carried out using the multipoint tool in ImageJ for a minimum of three images per sample set, and the following parameters were accounted for: TJ stand abundance was calculated as the sum of horizonal (HSI) and vertical intersections (VSI) of strands and lines. Mesh elongation was calculated as HIS/VSI, strand abundance was calculated as the product of the number of strand‐containing grid boxes and the box area of 0.01 µm^2^, strand density in a mesh of strands was calculated by dividing the stand abundance by the mesh area.

### Other Methodologies—Electrical Impedance Spectroscopy (EIS) and Analysis on BBB Spheroids

EIS on BBB spheroids was measured using a microcavity array chip that was provided and previously reported by Dr. Heinz‐Georg Jahnke from the University of Leipzig.^[^ ^39^ ^]^ The microcavity array chip consisted of pyramidal cavities with an edge length of 300–400 µm and depth 100 µm thereby, allowing the spheroids to sit well within the cavity. Firstly, the array was washed with 1 mL 70% EtOH and followed by rinsing with 1 mL PBS^−^. The arrays were then filled with 1 mL pre‐warmed EC+ medium and verified microscopically for the presence of bubbles. EIS was then measured and monitored using a previously developed multiplexer system and high precision impedance analyzer ISX‐3 from Sciospec Scientific Instruments, Germany.^[^ ^67^, ^68^ ^]^ Impedance spectra were recorded from 5 kHz to 5 MHz (51 points, 100 mVt amplitude). EIS was first recorded for a blank cavity, which did not contain any spheroid, followed by measurements of a minimum of 15 spheroids per sample condition. Raw data was then analyzed and processed with previously developed IDAT v3.6 software.^[^ ^39^ ^]^ Relative impedance or extracted cell signals was calculated automatically by the software using the following equation.

(1) $$Relativeimpedance\;\%=\frac{\left|Z\right|spheroid-\left|Z\right|blank}{\left|Z\right|blank}\times 100\%$$

|Z| blank [Ω] = Impedance value specific to blank cavity and |Z| spheroid [Ω] = Impedance value specific to cavity with spheroids.

### Other Methodologies—Small Molecule Tracer Permeability in Spheroids

Investigation of small molecule tracer flux was performed using one complete well (300 spheroids) containing iBCECs and control spheroids containing only ACs and PCs. In this set up, in comparison to standard cell culture inserts, the spheroids are considered as the “basolateral compartment” and the medium surrounding them is considered the “apical compartment”. Cell culture medium was first gently removed from the AggreWell 800 microwell containing spheroids and 1 mL of 10 µm Sodium Fluorescein (NaF) solution was added very carefully, by pipetting onto the walls of the microwell with minimum displacement of the spheroids. The plate was then placed into the incubator for 1 h at 37 °C. Sampling was performed post incubation time, 200 µL of 10 µm NaF solution (donor sample, time = 0 min), 200 µL of transport medium/blank medium, and 200 µL of NaF solution from the “apical compartment” were pipetted as triplicates into a black 96‐well plate for fluorescence measurements. Measurements were then performed with a fluorescence reader (TECAN Infinite M200) with the following boundary conditions. 2 × 2 multiple measurements (circle) per well, frame 500 µm, excitation bandwidth 9 nm, emission bandwidth 20 nm, number of flashes 25, integration time 20 µs, deceleration and rest time 0 µs, excitation wavelength 490 nm, emission wavelength 525 nm, gain 57. In order to calculate permeability, relative fluorescence unit (RFU) values of test conditions were first subtracted from the blank values and then normalized to donor concentrations.

### Other Methodologies—BBB Spheroid Histology and Immunocytochemistry

For standard spheroid slice stainings, a minimum of 20–30 spheroids were first collected in a 15 mL falcon tube and washed three times with PBS^−^. They were then fixed with 1 mL of 4% Roti‐Histofix at RT for 15 min. Fixed spheroids were washed once with PBS^−^ and embedded directly. To begin with, spheroids were carefully placed down onto a clean microscopy glass slide; excess mount of PBS^−^ was removed gently using a 10 µL pipette. Fifty microliters of molten Richard‐Allan Scientific HistoGel was then pipetted on top of the spheroids very gently, such that they all elevated and located at the same horizonzal plane. Once the gel solidified an additional 100 µL of molten Richard‐Allan Scientific HistoGel was added on top. Post gel solidification, the whole construct was fixed again with 1 mL of 4% Roti‐Histofix at RT for 15 min. The gel construct containing the spheroids was placed directly into an embedding cassette that included a filter paper. The closed cassette was placed into the embedding machine, and the paraffin embedding protocol as described in (**Table** **6**) was performed. After embedding, the samples were removed from the embedding cassette and placed onto a metal sample holder. The holder was filled with fresh molten paraffin in order to prepare a block, which could be cut by aid of a microtome. Five micrometers thick sections of the samples were prepared and collected onto a PLL coated microscopy slide and left in an oven at 37 °C overnight for drying. For analysis of different tissue structures, Hematoxylin and Eosin staining (H&E) (**Table** **7**) or immunohistochemistry (**Table** **8**) was performed.

**Table 6:** Steps followed in tissue paraffin embedding.

Step	Solution	Duration [h]
Washing	Deionized H_2_O	1
Dehydration (ascending alcohol series)	Ethanol 50% Ethanol 70% Ethanol 80% Ethanol 96% Isopropanol 1 Isopropanol 2 1:2 Isopropanol‐Xylene mixture	1 1 1 1 1 1 1
Removal of alcohol	Xylene 1 Xylene 2	1 1
Paraffin embedding	Paraffin 1 Paraffin 2	1.5 1.5

**Table 7:** Steps followed in H&E staining.

Step	Solution	Duration
Deparaffinization and rehydration	Xylene 1 Xylene 2 Ethanol 96% Ethanol 96% Ethanol 70% Ethanol 50% Deionized H_2_O	30 s 30 s 1 dip 1 dip 1dip 1 dip 1 dip
Staining of cell nuclie	Heamatoxylin	6 min
Washing	Deionized H_2_O	4 dips (till color runs out)
Staining of cytoplasm and ECM	Eosin	6 min
Washing	Deionized H_2_O	4 dips (till color runs out)
Draining	Ethanol 70% Ethanol 96% Isopropanol 1 Isopropanol 2 Xylene 1 Xylene 2	1 dip 1 dip 30 s 30 s 30 s 30 s
Embedding	Rapid mounting medium Entellan	Overnight, RT

**Table 8:** Steps followed in immunohistochemistry.

Step	Solution	Duration
Deparaffinization and Rehydration	Xylene 1 Xylene 2 Ethanol 96% Ethanol 96% Ethanol 70% Ethanol 50% Deionized H_2_O	30 s 30 s 1 dip 1 dip 1dip 1 dip 1 dip
Heat‐mediated antigen retrieval	Boiling in 10x Citrate buffer (pH 6)	8 min
Marking with hydrophobic PAP pen	‐	‐
Washing	PBS^−^ + 0.5% Tween‐20	1 dip
Blocking	PBS^−^ + 0.5% TritonX + 5% donkey serum	20 min
1° Antibody	1° Antibody in antibody dilution solution	Overnight,4 °C
Washing	PBS^−^ + 0.5% Tween‐20	3 ×5 min each
2° Antibody	2° Antibody in antibody dilution solution	2 h, RT
Washing	PBS^−^ + 0.5% Tween‐20	3 ×5 min each
Embedding	Mounting medium Fluoromount‐G^TM^ + DAPI	Overnight, RT

### Other Methodologies—BBB Spheroid Whole Mount Immunostaining

Immunofluoresence stainings were used to analyse the expression of relevant proteins in 3D using fluorochrome‐labeled antibodies. A minimum of ≈20–30 spheroids per sample were first collected into a 15 mL falcon and rinsed once with PBS^−^. The spheroids were then fixed with 500 µL ice cold Methanol:Acetone (1:1) for 5 min at RT or 500 µL 4% Roti‐Histofix at RT for 30 min depending on the antibody used. Post fixation, the cells were washed once with 500 µL PBS^−^ and stored in PBS^−^ at 4 °C for a maximum of 2 days until staining was performed (**Table** **9**). Stained spheroids were embedded into 96 well glass bottom plates and visualized using a Confocal SP8 microscope.

**Table 9:** Steps followed in 3D whole mount immunofluorescence staining.

Step	Solution	Duration
Permeabilization	PBS^−^ + 0.5% TritonX	30 min
Washing	PBS^−^	3 ×5 min each
Blocking	PBS‐ + 0.5% TritonX + 10% donkey serum	30 min
1° AB	1° Antibody in Antibody dilution solution	Overnight, 4 °C
Washing	PBS^−^	3 ×5 min each
2° antibody + Nuclear staining	2° Antibody + SYTOX red nuclear stain in Antibody dilution solution	Overnight, 4 °C
Washing	PBS^−^	3 ×5 min each
Embedding	Mounting medium Fluoromount‐G^TM^	Overnight

### Other Methodologies—Microscopy

Histological samples stained with H&E or with fluorochromes were visualized using the inverse fluorescence microscope BZ‐9000 (Keyence). Whole mount stained samples were visualized with the confocal microscope TCS SP8 (Leica Microsystems).

### Statistical Analysis

Data was analyzed using either Microsoft Excel Microsoft 365 MSO (Version 2310 Build 16.0.16924.20054) 64‐bit or GraphPad Prism 9 9.4.1 software. Unless otherwise specified all data are presented as mean ± standard deviations (SD) or standard error of mean (SEM). Transcriptomic analysis was performed utilizing the 2^‐∆∆CT method, and statistical significance was assessed using paired two‐tailed t‐Test. For additional experimental analyses, ordinary one‐way ANOVA was employed alongside Tukey's or Dunnett's multiple comparison tests. An alpha level of 0.05 was established as the threshold for significance. Results are presented with asterisks indicating the following significance levels: *p* ≤ 0.0001 = ^****^, *p* ≤ 0.001 = ^***^, *p* ≤ 0.01 = ^**^, *p* ≤ 0.05 = ^*^, and *p* > 0.05 designates a nonsignificant result (ns).

## Conflict of Interest

The authors declare no conflict of interest.

## Author Contributions

A.A.M. and M.M. performed conceptualization. S.M.S., E.R., S.G.,A.H. J.P., H.G.J, A.B., and W.N. performed methodology. S.M.S., E.R., and S.G. performed investigation. S.M.S. wrote the original draft. A.A.M, M.M., W.N., A.B., J.P., H.G.J, and S.O. wrote review and editing. M.M. and A.A.M. acquired funding and resources. M.M., A.A.M., and S.O. performed supervision.

## Supporting information



Supporting Information

## Data Availability

The data that support the findings of this study are available from the corresponding author upon reasonable request.
