# Supplementary material for: Generation of Advanced Blood–Brain Barrier Spheroids Using Human‐Induced Pluripotent Stem Cell‐Derived Brain Capillary Endothelial‐Like Cells
Source: Adv Biol (Weinh). 2025 Feb 6;9(4):2400442. doi: 10.1002/adbi.202400442 (PMC12001013; doi:10.1002/adbi.202400442)
Supplement: Supplementary file 1 — Supporting Information [file ADBI-9-2400442-s001.pdf]

ADVANCED BIOLOGY

Supporting Information

for *Adv. Biology*, DOI 10.1002/adbi.202400442

Generation of Advanced Blood–Brain Barrier Spheroids Using Human-Induced Pluripotent Stem Cell-Derived Brain Capillary Endothelial-Like Cells

*Sanjana Mathew-Schmitt, Sabrina Oerter, Evelin Reitenbach, Sabine Gätzner, Alevtina Höchner, Heinz-Georg Jahnke, Jörg Piontek, Winfried Neuhaus, Andreas Brachner, Marco Metzger and Antje Appelt-Menzel**

Supporting information

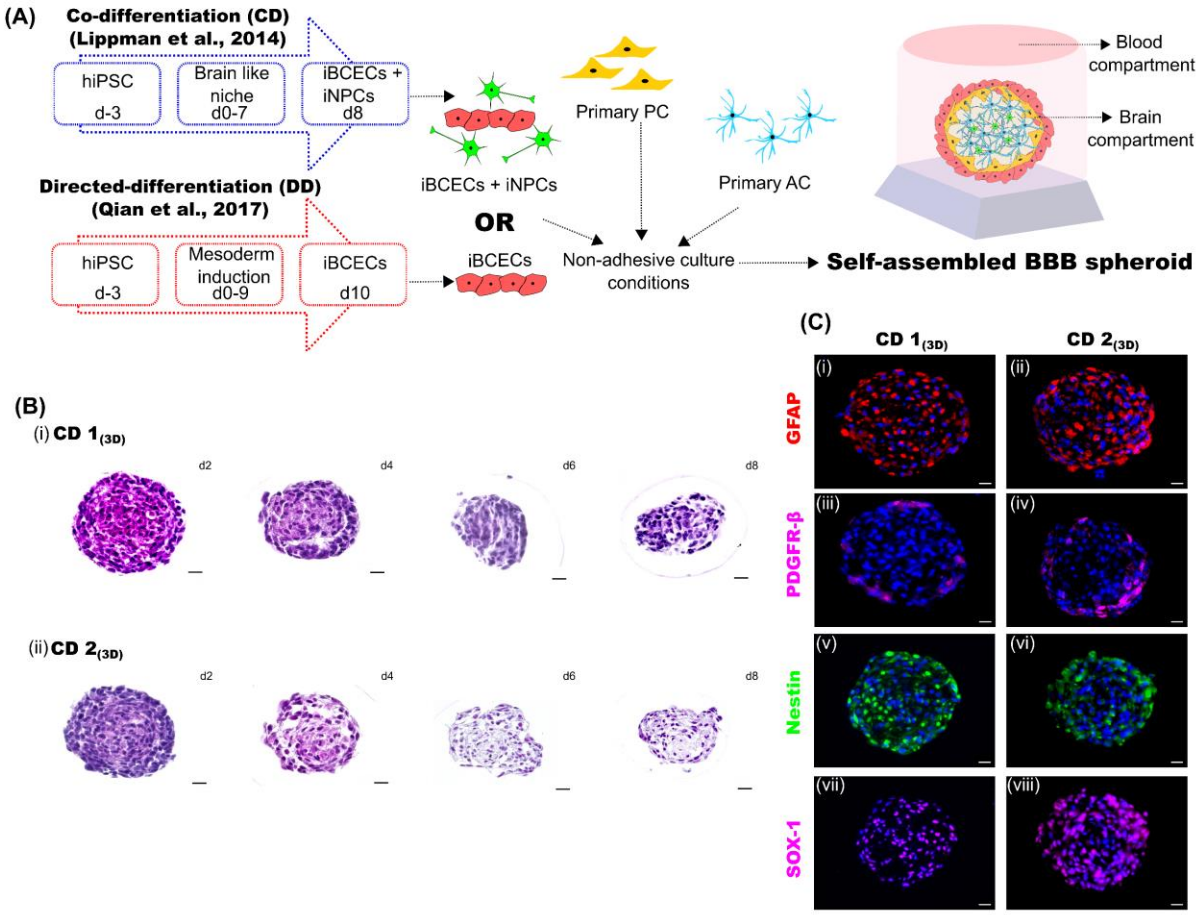

Figure S1: Generation, longevity and inclusion of neurovascular cell types in blood-brain barrier spheroids

Human induced pluripotent stem cell (hiPSC)-derived brain capillary endothelial-like cells (iBCECs) and neural progenitors (iNPC) on day 8 (d8) of differentiation from the co-differentiation (CD) strategy and iBCECs on d10 of differentiation from the directed differentiation (DD) strategy were pooled together with primary astrocytes (AC) and pericytes (PC) in order to form blood-brain barrier (BBB) spheroids. Pooled cells in respective cellular ratios were cultivated on top of non-adhesive cell culture surfaces, the cells further self-assembled to form spheroids which were used for downstream applications, after 2 days of formation **(A)**. Generated BBB spheroids were investigated for their compactness as well as longevity in culture. Hematoxylin & Eosin staining of 5 µm slices of BBB spheroids, over a cultivation time of 8 days (d2 – d8) indicate that spheroid morphology is maintained only up to a maximum of 2 days. Scale bar = 50 µm **(B, i-ii).** BBB spheroids were investigated for the expression of neurovascular unit cell types. Paraffin sections of 5 µm thickness were stained for AC marker Glial fibrillary acidic protein (GFAP) **(C, i-ii),** PC marker platelet-derived growth factor receptor beta (PDGFR-β) **(C, iii-iv),** and neural progenitor markers nestin **(C, v-vi)** and SRY-box transcription factor-1 (SOX-1) **(C, vii-viii)**. Microscopic images were captured at 20x magnification using the Keyence microscope. Scale bar = 20 µm. Nuclei were labelled with 4′, 6-diamidine-2-phenylindole (DAPI) in blue. N= 3 biological replicates.

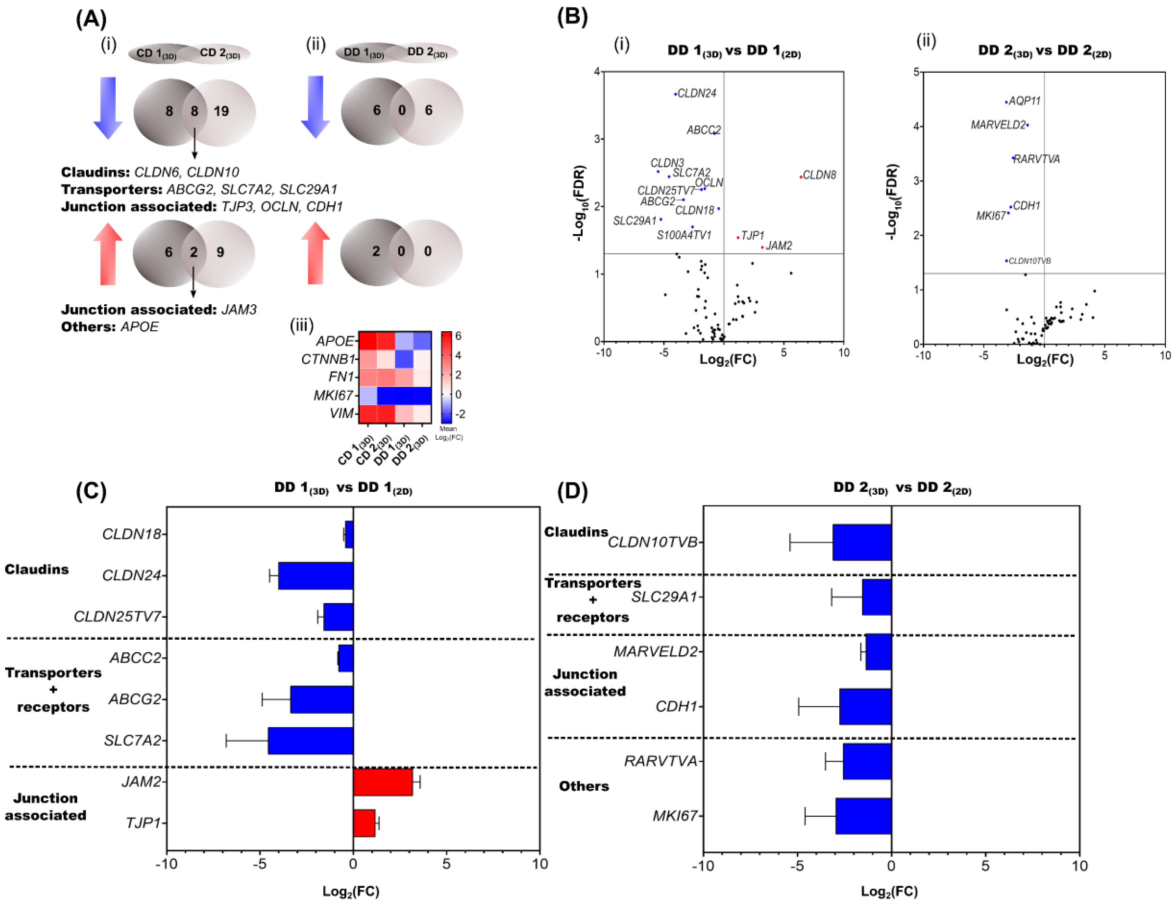

Figure S2: Higher expression of blood-brain barrier relevant transcripts is observed in spheroids containing iBCECs derived via co-differentiation (related to figure 1)

Relative gene expression of relevant blood-brain barrier (BBB) transcripts in BBB spheroids were obtained via high-throughput multiplex quantitative real time-polymerase chain reactions. Analysis was performed for a minimum of n = 3 (exceptions n = 2 for CD $1_{(3D)}$, DD $1_{(2D)}$) biological replicates per condition using the $2^{-\Delta\Delta Ct}$ method with mono-culture iBCECs cultivated on cell culture inserts as a reference. Mean Log_2 (Fold change, FC) are represented in the figure and p values ≤ 0.05 were considered significant. The total number of genes that are commonly regulated between BBB spheroids based on different differentiation strategies are represented in Venn diagrams **(A, i-ii)**. Heat map showing relevant mean Log_2 (FC) values are represented in **(A, iii)**. Volcano plots represent differentially up (red) and downregulated (blue) genes in DD. Top differentially regulated genes are labelled **(B, i-ii)**. Significantly regulated transcripts in DD $1_{(3D)}$ **(C)**, DD $2_{(3D)}$ **(D)** samples in comparison to DD $1_{(2D)}$ and DD $2_{(2D)}$ respectively are presented as bar graphs. Statistical significances were determined by paired two-tailed t-Test. Significantly regulated genes represent mean fold gene expression ± SD.

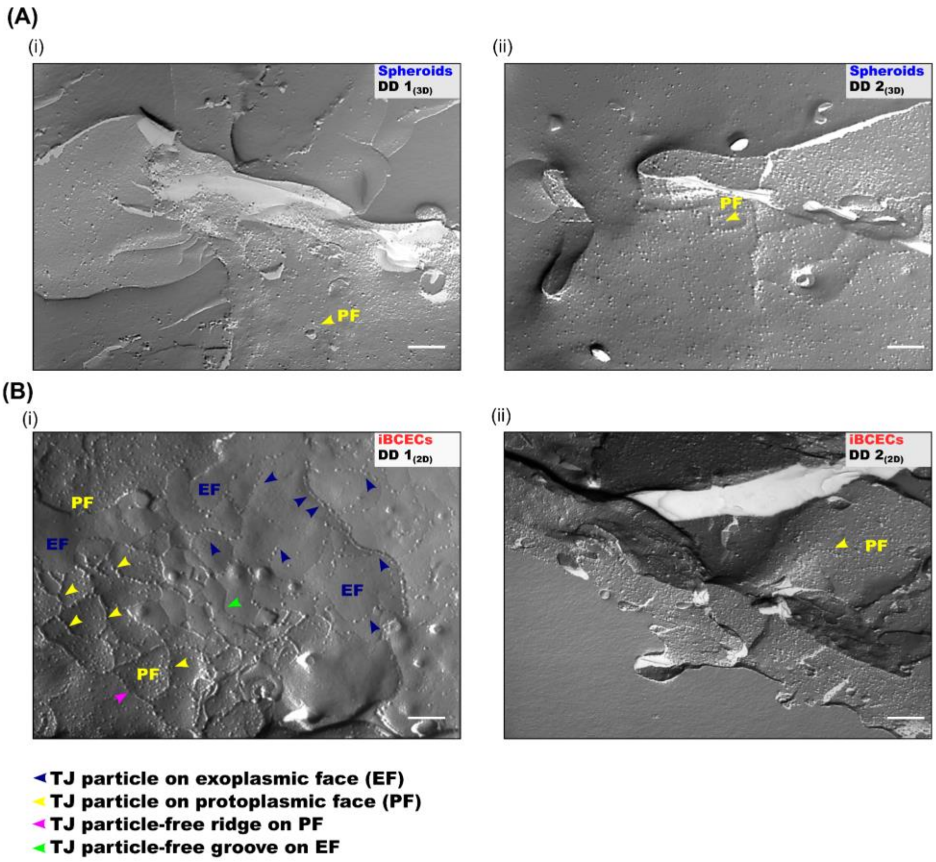

Figure S3: Freeze fracture electron micrographs of directed differentiation samples (related to figure 2)

Freeze fracture electron micrographs (FFEM) of samples generated using human induced pluripotent stem cell (hiPSC)-derived brain capillary endothelial-like cells (iBCECs) derived via directed differentiation (DD) captured at 50000x (A, B). Blue arrowheads indicate particles in the grooves on the exoplasmic face(E-face). Yellow arrowheads indicate TJ particles/fibrils on the protoplasmic face (P-Face). Pink arrowhead indicates TJ particle-free ridges on the P-Face and green arrowhead indicates a TJ particle-free groove on the E-Face. Images were captured for a minimum of n = 3 biological replicates per condition. Scale bar = 200 nm.

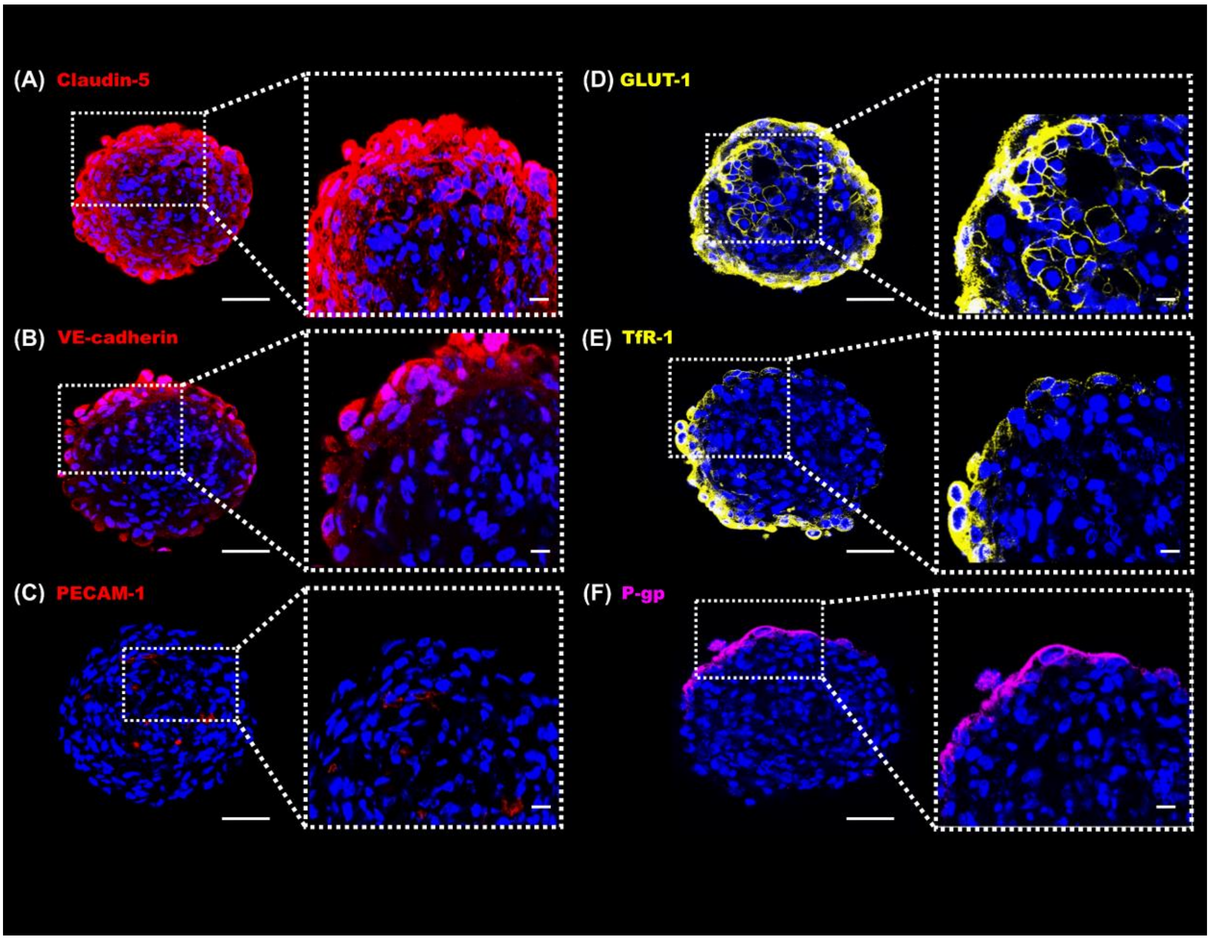

Figure S4: Immunofluorescence reveals tissue-specific protein expression (related to figure 4)

CD $2_{(3D)}$ samples were investigated for the expression of key brain capillary endothelial cell (BCEC) markers in n = 3 biological replicates. Images were captured at 40x magnification through a maximum Z stack capacity of the confocal laser scanning microscope and representative slice images of depth ranging from ~50µm - ~70µm are presented via single Z stacks (Scale bar = 50 µm). Zoomed in areas are indicated on the right panel of each spheroid (Scale bar = 20 µm). Nuclei were labelled with Sytox™ Red dead cell stain. Expression and localization of junctional proteins claudin-5 **(A)**, vascular endothelial-cadherin **(B)** and platelet endothelial cell adhesion molecule-1 **(C).** Expression of transport and receptor markers glucose transporter-1 **(D)**, transferrin receptor-1 **(E)** and P-glycoprotein **(F)** were verified in blood-brain barrier (BBB) spheroids.

Table S1: List of selected genes included in heat map (related to figure 1)

Symbol	Target	NM accession number	Mean $Log_2(FC) \pm SD$ CD $1_{(3D)}$	t-test P value	Mean $Log_2(FC) \pm SD$ CD $2_{(3D)}$	t-test P value
ABCA1	ATP binding cassette subfamily A member 1	NM_005502.4	4.783 ± 1.695	0.289	3.786 ± 0.164	≤ 0.0001
ABCA7	ATP binding cassette subfamily A member 7	NM_019112.4	0.754 ± 0.337	0.127	-0.963 ± 0.448	0.006
ABCB1	ATP binding cassette subfamily B member 1	NM_000927.4	0.961 ± 2.230	0.478	0.934 ± 0.372	0.036
ABCC1	ATP binding cassette subfamily C member 1	NM_004996.3	3.148 ± 0.996	0.185	0.828 ± 0.105	≤ 0.01
ABCC2	ATP binding cassette subfamily C member 2	NM_000392.4	1.621 ± 0.041	≤ 0.0001	-1.222 ± 0.726	0.020

ABCC4	ATP binding cassette subfamily C member 4	NM_005845.4	2.357 ± 0.532	0.086	1.567 ± 0.822	0.061
ABCC5	ATP binding cassette subfamily C member 5	NM_005688.3	1.621 ± 0.118	0.007	-1.656 ± 0.157	≤ 0.0001
ABCG2	ATP binding cassette subfamily G member 2	NM_004827.2	-5.220 ± 0.937	≤ 0.0001	-6.067 ± 2.393	≤ 0.0001
SLC2A-1	Solute carrier family 2 member 1	NM_006516.2	0.620 ± 1.086	0.468	0.552 ± 0.533	0.186
SLC7A1	Solute carrier family 7 member 1	NM_003045.4	1.451 ± 1.089	0.297	0.831 ± 1.487	0.248
SLC7A2			-1.623 ± 0.830	0.041	-3.273 ± 1.874	≤ 0.01
SLC16A1	Solute carrier family 16 member 1	NM_003051.3, NM_001166496.1	0.078 ± 1.446	0.722	-0.927 ± 0.524	0.021

SLC16A2	Solute carrier family 16 member 2	NM_006517.4	1.926 ± 1.106	0.259	0.299 ± 0.460	0.319
SLC29A1	Solute carrier family 29 member 1	NM_001078175.2, NM_001078177.1, NM_001304462.1	-1.482 ± 0.839	0.056	-2.287 ± 1.245	0.005
MFSD2A-II	Major facilitator superfamily domain containing 2A	NM_001349821.1, NM_001349823.1, NM_001349822.1, NM_032793.4, NM_001136493.2, NM_001287809.1,	3.061 ± 1.423	0.272	2.698 ± 0.487	0.008
CLDN1	Claudin 1	NM_021101.5	6.766 ±1.779	0.292	6.006 ± 4.081	0.265
CLDN4	Claudin 4	NM_001305.4	-1.353 ± 1.498	0.253	-1.451 ± 1.792	0.453
CLDN5	Claudin 5	NM_001130861.1, NM_003277.3	0.926 ± 1.032	0.370	1.392 ± 0.579	0.063
CLDN6	Claudin 6	NM_021195.4	-2.041 ± 0.719	0.013	-3.555 ± 1.568	≤ 0.01
CLDN7	Claudin 7	NM_001307.5	-0.831 ± 0.664	0.159	-1.973 ± 1.386	0.036
CLDN8	Claudin 8	NM_199328.2	-6.970 ± 1.911	≤ 0.0001	-3.494 ± 1.899	0.002
CLDN10 TVB	Claudin 10	NM_006984.4	-1.776 ± 0.650	0.018	-3.083 ± 2.88	0.010
CLDN11	Claudin 11	NM_005602.5	4.309 ± 1.670	0.289	3.835 ± 1.563	0.094
CLDN12 TV 1	Claudin 12	NM_001185072.2	2.384 ± 3.244	0.420	1.630 ± 1.311	0.246
CLDN12 TV 2	Claudin 12	NM_001185073.2	1.536 ± 1.206	0.314	0.250 ± 0.817	0.518
CLDN12TV 3	Claudin 12	NM_012129.4	1.508 ± 1.854	0.404	0.471 ± 1.010	0.371
CLDN15	Claudin 15	NM_014343.2, NM_001185080.1	0.439 ± 0.254	0.163	-0.423 ± 1.952	0.788

CLDN24	Claudin 24	NM_001185149.1	3.118 ± 0.396	0.043	0.388 ± 2.747	0.432
CLDN25 TV1	(putative) Claudin 25 /	NM_001101389.1	1.569 ± 0.914	0.240	0.589 ± 0.867	0.218
CLDND1 TV1 CLDND1 TV6	ClaudinD1	NM_001040181.1; NM_001040199.1	-0.170 ± 0.872	0.947	-1.157 ± 0.970	0.044
JAM1	Junctional adhesion molecule 1	NM_016946.4	-1.380 ± 1.088	0.120	-2.106 ± 1.251	0.011
JAM2	Junctional adhesion molecule 2	NM_021219.3	6.035 ± 0.700	0.096	4.945 ± 1.117	0.112
JAM3	Junctional adhesion molecule 3	NM_032801.4	1.644 ± 0.337	0.052	1.689 ± 0.591	0.046
TJP1	Tight junction protein 1	NM_003257.4	0.658 ± 0.599	0.302	0.084 ± 0.713	0.639
TJP2	Tight junction protein 2	NM_001170414.2, NM_201629.3, NM_001170416.1, NM_001170415.1, NM_004817.3	-0.710 ± 0.459	0.114	-2.043 ± 1.103	0.008
TJP3	Tight junction protein 3	NM_001267561.1	-3.316 ± 0.832	0.002	-4.409 ± 1.543	≤ 0.0001
OCLN	Occludin	NM_001205255.1, NM_001205254.1, NM_002538.3	-1.361 ± 0.375	0.0138	-1.851 ± 0.772	0.001

MARVELD2	MARVEL domain containing 2	NM_001244734.1, NM_001038603.2	-1.292 ± 0.805	0.077	-1.560 ± 0.385	≤ 0.001
CDH1	Cadherin 1	NM_001317186.1, NM_001317185.1, NM_001317184.1, NM_004360.4	-4.134 ± 2.185	0.006	-3.512 ± 1.659	≤ 0.001
CDH5	Cadherin 5	NM_001795.4	-0.819 ± 3.863	0.664	-0.579 ± 3.115	0.546
PECAM1	Platelet endothelial cell adhesion molecule 1	NM_000442.5	6.038 ± 1.314	0.224	6.128 ± 1.742	0.209
CTNNB1	Catenin beta-1	NM_001098209.1, NM_001904.3, NM_001098210.1	2.541 ± 0.616	0.104	0.8111 ± 0.701	0.144
VIM	Vimentin	NM_003380.4	5.512 ± 1.632	0.276	5.616 ± 0.621	0.022
FN1	Fibronectin	NM_212474.2, NM_212476.2, NM_212478.2, NM_002026.3, NM_212482.2, NM_001306132.1, NM_001306131	3.087 ± 1.968	0.342	3.348 ± 1.543	0.089
APOE	Apolipoprotein E	NM_000041.4, NM_001302688.2, NM_001302689.2, NM_001302690.2, NM_001302691.2	0.577 ± 0.120	0.030	-1.827 ± 0.628	≤ 0.001
LRP1	LDL receptor related protein 1	NM_002332.2	2.212 ± 0.207	0.016	1.794 ± 0.468	0.020

LRP8	LDL receptor related protein 8	NM_004631.4, NM_001018054.2, NM_033300.3, NM_017522.4	1.535 ± 1.304	0.332	0.737 ± 0.667	0.124
RAGE	Receptor for advanced glycation endproducts	NM_001136.5, NM_001206929.2, NM_001206932.2, NM_001206934.2, NM_001206936.2, NM_001206940.2, NM_001206954.2, NM_001206966.2, NM_172197.3	1.840 ± 0.490	0.090	-2.915 ± 0.295	≤ 0.0001
INSR	Insulin receptor	NM_001079817.2, NM_000208.3	0.573 ± 0.220	0.090	-0.277 ± 0.908	0.864
TFRC	Transferrin receptor protein 1	NM_001313966.1, NM_001313965.1, NM_003234.3, NM_001128148.2	1.332 ± 1.613	0.398	0.539 ± 0.945	0.259
VEGFA	Vascular endothelial growth factor A	NM_001204384.1, NM_001171622.1, NM_001033756.2, NM_001025370.2, NM_001025369.2, NM_001025368	6.395 ± 0.742	0.105	5.583 ± 0.600	0.017
RARA TVA	Retinoic acid receptor alpha	NM_000964.4, NM_001024809.4, NM_001145301.3, NM_001145302.3	1.00 ± 0.392	0.114	-0.919 ± 1.024	0.190

RARB TVB	Retinoic acid receptor beta	NM_000965.4, NM_016152.3, NM_001290277.1	1.978 ± 0.684	0.143	-1.705 ± 1.541	0.085
RXRA TV1	Retinoic acid receptor RXR-alpha	NM_001291920.2, NM_001291921.2, NM_002957.6	2.719 ± 0.626	0.102	0.733 ± 1.201	0.321
RXRB TV2	Retinoic acid receptor RXR-beta	NM_001270401.2, NM_001291989.2, NM_021976.5	1.853 ± 0.479	0.087	0.917 ± 0.912	0.209
MKI67	Marker of proliferation Ki-67	NM_001145966.1, NM_002417.4	-0.831 ± 2.272	0.952	-2.970 ± 1.562	0.003
